# KASH proteins transform from passive tethers to dynamic conductors of motor-driven nuclear dynamics

**DOI:** 10.1016/j.ceb.2025.102578

**Published:** 2025-08-10

**Authors:** G. W. Gant Luxton, Selin Gümüşderelioğlu, Kassandra M. Ori-McKenney, Daniel A. Starr, Richard J. McKenney

**Affiliations:** Department of Molecular and Cellular Biology, University of California, 1 Shields Ave, Davis, CA 95616, United States

## Abstract

Nuclear-cytoskeletal coupling orchestrates critical cellular processes from migration to tissue organization. At the core of this machinery, outer nuclear membrane Klarsicht/ANC-1/SYNE homology (KASH) proteins function as sophisticated molecular conductors rather than simple structural tethers. This review examines three principles redefining these versatile proteins: specialized interfaces for selective microtubule motor protein recruitment that orchestrate diverse chromosomal and nuclear dynamics, coordination of multiple cytoskeletal systems through simultaneous engagement with actin and microtubules, and tissue-specific regulation that explains the diverse KASH protein-related disease manifestations. This framework provides insights into conditions from muscular dystrophy to neurodegeneration and suggests targeted therapeutic opportunities.

## Introduction

Nuclear-cytoskeletal coupling underlies diverse cellular functions including migration, division, meiotic chromosome pairing, and tissue morphogenesis, with defects in this process linked to various pathological conditions [1—4]. KASH proteins, once viewed as passive mechanical linkers, have now been revealed as sophisticated conductors of cytoskeletal dynamics. These outer nuclear membrane proteins contain a characteristic membrane-spanning C-terminal KASH domain [5] and typically function within the linker of nucleoskeleton and cytoskeleton (LINC) complex alongside inner nuclear membrane Sad1/UNC-84 (SUN) proteins, enabling transmission of cytoskeletal forces to the nucleus and chromosomes [6,7].

**Figure 1:**
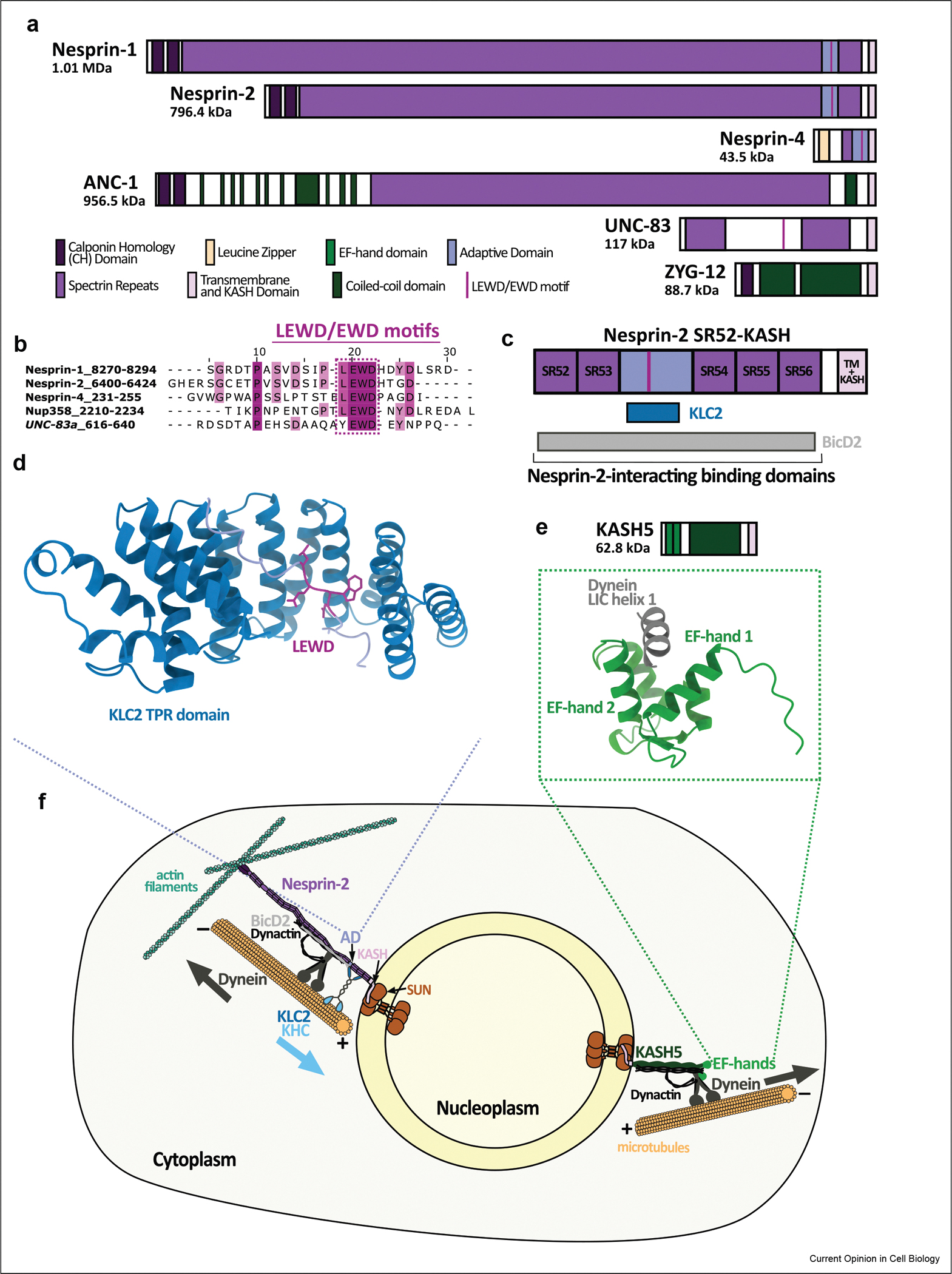
Molecular Architecture and Regulation of KASH protein-Motor Interactions. **a)** Structural organization of select KASH protein family members showing domain composition across species, including mammalian Nesprins, *C. elegans* ANC-1, UNC-83, and ZYG-12, illustrating evolutionary conservation and functional specialization. The AD (dark blue) is highlighted as a critical hub for motor interactions, with LEWD/EWD motifs (red) indicated. **b)** Alignments of LEWD/EWD motifs (with conserved residues highlighted in blue) showing sequence conservation in Nesprin-1, -2, -4, Nup358, and UNC-83. **c)** Schematic representation of Nesprin-2’s C-terminus domain organization highlighting SR52–56 and their relationships to binding domains for KLC2 and BicD2. **d)** AlphaFold3 predicted structure of kinesin light chain 2 (KLC2) TRP domain interaction with the LEWD motif (highlighted in blue) of *C. elegans* UNC-83. **e)** AlphaFold3 predicted structure of KASH5 EF-hand domains (green) interacting with dynein light intermediate chain (LIC) helix 1 (grey) [12]. **f)** Integration of KASH protein activities at the nuclear envelope, showing both Nesprin-2G and KASH5 systems. Nesprin-2 simultaneously engages both kinesin-1 (via KLC2) and the dynein/dynactin/BicD2 complex to coordinate bidirectional transport along microtubules while also engaging actin filaments through its N-terminal CH domains. KASH5 interacts with dynein through its specialized EF-hand domains. Together, these specialized interactions demonstrate how different KASH proteins have evolved to fulfill distinct roles: Nesprin-2 in nuclear positioning during development and migration, while KASH5 mediates telomere movements during meiotic chromosome pairing.

This review explores recent advances in our understanding of nuclear-cytoskeletal coupling, highlighting how mechanistic molecular insights reshape our understanding of development and disease. Three key principles have transformed our understanding (Figure 1): (1) specific molecular mechanisms govern microtubule motor protein recruitment and activation by KASH proteins, with distinct structural motifs providing specialized interfaces for motor binding; (2) KASH proteins coordinate bidirectional transport through simultaneous engagement of opposite-polarity motors, with specific KASH proteins like Nesprin-2G recruiting both kinesin-1 directly and dynein via BicD2 (Figure 1f); and (3) these activities are tuned to tissue-specific mechanical and developmental contexts, explaining the diversity of phenotypes in KASH protein-related diseases [8*]. Each principle represents a paradigm shift in our understanding of nuclear-cytoskeletal dynamics.

## Molecular mechanisms of motor recruitment and stoichiometry

The identification of specific molecular interfaces for microtubule motor protein recruitment has fundamentally transformed our understanding of KASH protein function. KASH5 exemplifies this breakthrough as a novel transmembrane dynein activating adaptor [9**,10*]. Through specialized coiled-coil (CC) and EF-hand domains, KASH5 directly interacts with dynein by acting as one of a growing family of adapter proteins that links dynein to dynactin (Figure 1e) - not for general nuclear positioning, but for telomere movements during meiotic chromosome pairing [11]. Despite this specialized function, KASH5 shares key architectural features with nuclear-positioning KASH proteins: its transmembrane structure localizes dynein activation to specific membrane sites, and its calcium-insensitive EF-hand domains functionally resemble other specialized dynein adaptors [12,13]. This evolutionary specializing of motor interfaces exemplifies how the KASH protein family has diversified to control different nuclear and chromosomal movements.

The W-acidic motif, often comprising the amino acid sequence LEWD [14,15], represents another specialized motor-binding interface within the adaptive domain (AD) of several KASH proteins [16,17] (Figure 1d). The AD is a functionally critical region mediating interactions with cytoskeletal components and serving as a hub for motor protein recruitment. This LEWD motif directly binds kinesin light chain (KLC), while sequences immediately adjacent to this region are known to bind to the dynein adapter BicD2 [18*], establishing KASH proteins as versatile cargo adaptors that can engage both kinesin-1 and dynein machinery. This dual-motor binding capability distinguishes KASH proteins from other adaptors that primarily interact with single motor systems [19,20]. The W-acidic motif binds within the tetratricopeptide repeat (TPR) domains of the KLCs (Figure 1b—d), potentially aiding in motor activation [14,21*]. In Nesprin-2G, this interaction anchors centrosomes to nuclei [22], while Nesprin-1 positions nuclei in multinucleated muscle cells using this motif [16]. Mutations in W-acidic motif of Nesprin-4 disrupt nuclear positioning in outer hair cells, resulting in hearing loss [23,24]. Similarly, the *C. elegans* functional homolog of Nesprin-4, UNC-83, uses an EWD motif to mediate interactions between migrating nuclei and kinesin-1 [23], demonstrating the evolutionary conservation of this mechanism.

Beyond direct motor interactions as seen with KASH5, other KASH proteins, like Nesprin-2, engage dynein through adaptor-mediated interactions. The adaptor protein BicD2 facilitates dynein-dependent nuclear movements during brain development through direct interactions with Nesprin-2G [25]. The region of Nesprin-2G mediating this interaction may be near the kinesin-binding LEWD motif, potentially coordinating different motor activities [18*,^26^]. Recent work by Zhou et al. (2024) provides evidence that Nesprin-2G can simultaneously bind both kinesin-1 and BicD2 (along with dynein-dynactin), enabling coordinated bidirectional transport essential for precise nuclear positioning [27**]. Notably, this bidirectional movement shows a slight bias towards minus-end directed movement, potentially representing an important regulatory feature for controlling the overall direction of nuclear translocation.

Further investigation into the Nesprin-2-BicD2 interaction has revealed notable mechanistic details. Spectrin repeats 52—56 (SR52–56) of Nesprin-2 interact with BicD2 through a mechanism distinct from other dynein adaptors [28]. Yi et al. (2023) mapped this interaction, identifying amino acids 750—800 in the BicD2 CC3 domain as the critical binding region for Nesprin-2 [18*]. Interestingly, this region overlaps with, but is distinct from, the binding site for another BicD2 cargo, RanBP2. Sequence alignments reveal limited conservation between the BicD2-binding regions of Nesprin-2 and RanBP2, which elegantly explains how specific mutations in BicD2 can selectively disrupt binding to either cargo, resulting in distinct clinical manifestations [18*,^25^,^26^].

Evolutionary diversity exists in KASH-mediated motor recruitment. ZYG-12, a functional homologue of KASH5 *in C. elegans*, directly recruits dynein during meiosis, functioning as a Hook protein with a KASH domain [9**]. This suggests multiple solutions have evolved to achieve similar nuclear positioning outcomes across species, while maintaining key conserved interaction motifs. Beyond nuclear positioning proper, these motor-KASH protein interactions play broader roles in cellular organization, as exemplified by SUN2 and Nesprin-2G, which work with kinesin KIF20A to regulate Golgi apparatus organization in response to chromatin state changes [29].

An important consideration in these KASH-motor interactions is their stoichiometric relationship. The SUN-KASH interaction occurs with a 3:3 ratio, where a trimer of SUN proteins interacts with three KASH proteins [30]. However, the KASH5 coiled-coil domain is dimeric in isolation [9**], as is Nesprin-2G [31**], leaving questions about the structural organization of these complexes. This creates a fascinating structural puzzle when engaging with dimeric motor proteins like dynein and kinesin-1. Neighboring SUN-KASH complexes may resolve this mismatch by enabling the formation of functional dimers of KASH proteins that effectively engage with dimeric motor complexes. Future efforts towards resolving the structural organization of KASH-motor interactions are needed shed further light on this issue.

## Coordination of motor activities

KASH proteins may coordinate bidirectional transport by simultaneously engaging multiple cytoskeletal systems. Nesprin-2G exemplifies this coordination by interacting with both microtubule motors and actin filaments [31**]. This multivalent engagement raises several fundamental questions about how motor activities are balanced — how binding of one motor influences another’s activity and what determines directional preference during bidirectional movement.

### Integration of multiple motor systems

A single SUN protein homotrimer can interact with three different KASH proteins [32], possibly creating diversity in the cytoskeletal elements engaged by a single LINC complex. However, this raises interesting questions about how hetero-hexameric SUN-KASH complexes could accommodate dimeric motor proteins like dynein and kinesin-1. The AD functions as the command center for motor coordination with its intrinsic structural flexibility and regions of partial disorder [17,33] potentially enabling dynamic regulation of motor interactions in response to cellular needs (as discussed below in Biological Implementation).

### Cross-cytoskeletal coordination

Sahabandu et al. (2025) demonstrated that Nesprin-2G can simultaneously bind both kinesin motors via its LEWD motif, and actin filaments via its calponin homology (CH) domains [31**]. This coordination represents a specialized example of active cytoskeletal crosstalk, distinct from passive mechanisms [34]. Nesprin-2G couples kinesin-1 activity to actin organization, with MAP7D3 strongly enhancing this interaction. MAP7 family proteins are now recognized as critical regulators of kinesin activity [21*], [35—37] and play known roles in KASH-dependent nuclear movement in *Drosophila* muscle cells [38].

Intriguingly, this functional architecture has evolved independently across kingdoms of life. In plants, certain kinesin family proteins contain CH domains integrated directly into the tail domain of kinesin family motors [39—42], functioning during both interphase and cell division. Among metazoan proteins, ACF7/MACF1 represents another example with N-terminal tandem CH domains for actin binding and a capacity to interact with microtubule plus-ends, though through mechanisms distinct from direct kinesin engagement [43]. Similarly, fungi employ distinct mechanisms, with dynein anchored at the cell cortex to pull astral microtubules during nuclear migration in *S. cerevisiae* [44]. The parallel evolution of proteins that coordinate actomyosin and microtubule systems across diverse lineages underscores the fundamental importance of active cytoskeletal crosstalk for nuclear positioning and broader cellular organization.

### Regulatory mechanisms

The involvement of MAP7D3 in enhancing Nesprin-2G-kinesin-1-actin interactions [31**] represents an important regulatory layer in nuclear positioning. MAP7 family proteins contain unique domains that both interact with microtubules and help relieve kinesin-1 autoinhibition, serving as critical spatial regulators of motor activity [36,45*]. Metzger et al. [38] demonstrated that MAP7/Ensconsin is essential for nuclear positioning in *Drosophila* muscle cells, where it cooperates with kinesin-1 to drive proper nuclear spacing. The diverse MAP7 family members found across tissues [37,46] may contribute to the context-specific regulation of KASH protein-motor interactions, potentially explaining some of the tissue-specific phenotypes observed in KASH protein-related disorders [8*].

While most members of the MAP7 family recruit autoinhibited kinesin-1 to microtubules [21*,^35^—^37^], Sahabandu et al. revealed that MAP7D3 facilitated the formation of the Nesprin-2G-kinesin-1 complex even in the absence of microtubules [31**], revealing a new role for MAP7 proteins in stimulating kinesin-cargo interactions outside of its role on microtubules. Thus, MAP7 proteins may be master regulators of kinesin activity, dictating both the formation of kinesin-cargo complexes and recruitment of these complexes to microtubules. The regulation of these interactions likely occurs at multiple levels. Nuclear mechanosensing studies [47,48] have demonstrated that KASH proteins respond to mechanical forces at the nuclear envelope. Meanwhile, mechanistic work on motor complex assembly, particularly the studies by Garner et al. (2023) [10*] on KASH5-dynein interactions, has revealed distinct assembly steps that provide points for regulation.

## Biological Implementation

The molecular principles of KASH protein-motor regulation manifest differently across tissues, reflecting specialized cellular architectures. In neuronal development, bidirectional motility control through Nesprin-2G (Figure 1f) enables nuclei to navigate complex tissue environments while maintaining directed migration [27**]. The structural organization of Nesprin-2, with its actin-binding domain and motor-interacting region, provides a uniquely integrated platform for simultaneously engaging both the actin and microtubule cytoskeletons while precisely coordinating motor activities during complex nuclear movements. This coordination proves particularly important in cerebellar granule cells, where nuclear rotation and translocation determine proper neuronal positioning [49]. Similar mechanisms have been observed in *C. elegans*, where UNC-83 recruits both kinesin-1 and dynein to the nuclear envelope, enabling bidirectional movements and nuclear rolling that help bypass cytoplasmic obstacles during migration [50]. Pioneering studies demonstrated that while kinesin-1 provides the primary force for forward nuclear movement, dynein-mediated backward movements and rotational motions are critical for resolving encounters with cellular roadblocks.

### Nuclear dynamics in confined migration

KASH protein-mediated nuclear positioning faces unique challenges during confined migration, where cells must navigate through tight spaces that constrain nuclear deformation [51,52]. During transmigration through confined pores, the balance of forces between actin and microtubule systems becomes critical for successful nuclear passage [53]. Nesprin proteins can simultaneously engage both actin and microtubule motor systems [31**]. During confined migration, actomyosin contractility helps deform nuclei [54—56] while microtubule motors provide directional guidance [53,57,58]. Recent work has shown that nuclear envelope mechanics and LINC complex integrity are essential for cells navigating confined environments, where failure of nuclear-cytoskeletal coupling can lead to nuclear envelope rupture and DNA damage [59—62]. Different cell types have evolved distinct strategies, with immune cells positioning their nucleus frontward as a mechanical sensor to navigate the path of least resistance [53,63,64], while also dynamically adapting their nuclear architecture upon confinement *in vivo* to optimize deformation while preventing damage [65*]. Conversely, loss of LINC complex components increases cellular deformability [60], demonstrating the critical balance these proteins maintain in cellular mechanics during migration. This specialized coordination highlights how KASH proteins must adapt their motor regulation to meet the mechanical demands of different cellular contexts.

### Tissue-specific adaptations

Muscle tissue presents distinct challenges, requiring precise positioning of multiple nuclei along multinucleated fibers [3]. Nesprin-1α2, a muscle-specific splice form, plays a specialized role, with its kinesin interaction being essential for proper nuclear distribution [66]. Without this interaction, nuclear aggregation, and impaired muscle function result, highlighting how tissue-specific KASH protein isoforms adapt to mechanical environments.

### Disease manifestations

The importance of proper KASH protein function is underscored by their association with various human diseases [8*]. Mutations in Nesprin-1 and -2 cause Emery-Dreifuss muscular dystrophy (EDMD) [67], dilated cardiomyopathy [68], cerebellar ataxia [69] and a rare form of amyotrophic lateral sclerosis [70]. Many disease-causing mutations cluster within the AD [8*], suggesting disruption of KASH protein-motor interactions as a pathogenic mechanism [67,68]. Yi et al. (2023) demonstrated that mutations in BICD2 can selectively affect either RanBP2 or Nesprin-2 binding, leading to specific developmental defects in either radial glial progenitor cells or post-mitotic neurons, respectively [18*]. This selective disruption of cargo interactions provides a molecular explanation for how mutations in a single adaptor protein can result in distinct clinical presentations. In another example, mutations in Nesprin-1 can cause either muscular dystrophy or cerebellar ataxia, suggesting that different tissues have unique requirements for KASH protein function [8*]. Understanding these tissue-specific requirements will be essential for developing targeted therapeutic approaches.

## Future directions

Understanding the structure and regulation of the motor-binding AD stands as a key priority for future research. Despite its central role in motor recruitment and activation [14,15], many questions about this domain remain unanswered. High-resolution structural studies are needed to determine how the AD interacts with motors and how force, post-translational modifications, and cellular signals modulate these interactions.

### Methodological advances

New technical approaches will be essential for addressing these questions. Force-sensitive probes for monitoring mechanical interactions at the nuclear envelope [47,48], combined with optogenetic tools for controlling motor activity [71], will help reveal how KASH proteins coordinate multiple types of motors in response to mechanical cues. *In vivo*studies using these tools will be crucial for understanding how KASH-motor interactions are regulated during development and disease.

The integration of complementary methodologies presents particularly exciting opportunities. AlphaFold 3 predictions [72] could guide targeted cryo-electron tomography studies of intact LINC complexes, addressing fundamental questions about the three-dimensional organization of KASH protein-motor assemblies in their native membrane context. Recent successes combining these approaches to solve membrane protein complexes, such as the nuclear pore complex [73,74], provide a methodological template. Challenges remain in applying these techniques to the nuclear envelope’s curved geometry and the transient nature of motor-KASH protein interactions, but innovations in focused ion beam milling [75] offer promising solutions. Additionally, proximity labeling approaches like BioID or APEX [76] could map the dynamic interactome of KASH proteins in different cellular contexts, potentially revealing tissue-specific interaction partners that modulate motor activities.

### Therapeutic opportunities

The therapeutic implications of these advances are significant. Specific KASH protein-motor interaction interfaces, particularly within the AD, open possibilities for intervention in nuclear positioning diseases. For example, the LEWD motif that mediates kinesin-1 binding presents a discrete target for molecular intervention in EDMD, where Nesprin mutations disrupt nuclear positioning in muscle cells [16]. Interventions could work in several ways: (1) synthetic peptides containing optimized LEWD motifs could be delivered to strengthen weakened nesprin-kinesin interactions; (2) small molecules that stabilize these interactions could enhance nuclear transport; or (3) gene therapy approaches could restore functional LEWD motifs. Along these lines, Dodding and colleagues have pioneered “KinTag” peptides that mimic and enhance natural cargo—adaptor interactions with kinesin-1 [77]. These engineered peptides demonstrate significantly higher kinesin-binding affinity than natural LEWD-containing sequences and can effectively enhance cargo transport when genetically encoded. Related work with small molecules that modulate kinesin-cargo interactions has shown promise in controlling microtubule dynamics [78]. Similarly, the EF-hand domains of KASH5 [9**] could potentially be targeted to address meiotic defects. The high-resolution structures of these interaction interfaces (Figure 1d and e) provide a foundation for rational therapeutic design, while tissue-specific expression patterns of different KASH isoforms [79—81] offer a pathway to achieving the selectively essential for minimizing off-target effects — particularly important given the diverse roles of KASH proteins across different tissues and developing contexts.

## Conclusions

Recent discoveries have transformed our understanding of KASH proteins from passive tethers to dynamic conductors orchestrating nuclear-cytoskeletal coupling. The elucidation of specialized motor recruitment domains, cytoskeletal coordination mechanisms, and tissue-specific regulation provides a comprehensive framework for understanding nuclear dynamics in health and disease. These proteins exemplify nature’s elegant solution to a complex engineering challenge: how cells integrate mechanical and biochemical signals. KASH proteins illustrate the remarkable precision with which cells integrate mechanical and biochemical signals, orchestrating cytoskeletal systems to fulfill tissue-specific roles. This framework not only reshapes fundamental cell biology concepts but also illuminates new therapeutic strategies for KASH-related disorders.

The three principles outlined in this review — specialized motor recruitment interfaces, active coordination of cytoskeletal systems, and tissue-specific regulation — provide a roadmap for future investigations and therapeutic development. As structural biology techniques continue to advance, particularly with tools like AlphaFold 3 [72], we anticipate rapid progress in understanding the molecular details of KASH protein-motor interactions, potentially leading to novel interventions for diseases ranging from muscular dystrophies to neurodegenerative conditions.

## Data Availability

No data was used for the research described in the article.
